# Influential factors for medical students’ classroom concentration—evaluation with speech recognition and face recognition technology

**DOI:** 10.1186/s12909-024-06204-5

**Published:** 2024-10-31

**Authors:** Xiaohan Chai, Jingwen Yang, Yunsong Liu

**Affiliations:** Department of Prosthodontics, Peking University School and Hospital of Stomatology & National Center of Stomatology & National Clinical Research Center for Oral Diseases & National Engineering Research Center of Oral Biomaterials and Digital Medical Devices & Beijing Key Laboratory of Digital Stomatology & NHC Key Laboratory of Digital Stomatology & NMPA Key Laboratory for Dental Materials & Virtual Teaching and Research Section(VTRS) of Prosthodontics , 22 Zhongguancun South Avenue, Haidian District, Beijing, 100081 PR China

**Keywords:** Teaching strategies, Class concentration, Artificial intellengence, Speech recognition, Face recognition technology

## Abstract

**Statement of the problem:**

The concentration of medical students in the classroom is important in promoting their mastery of knowledge. Multiple teaching characteristics, such as speaking speed, voice volume, and question use, are confirmed to be influential factors.

**Purpose:**

This research aims to analyze how teachers’ linguistic characteristics affect medical students’ classroom concentration based on a speech recognition toolkit and face recognition technology.

**Materials and methods:**

A speech recognition toolkit, WeNet, is used to recognize sentences during lectures in this study. Face recognition technology (FRT) is used to detect students’ concentration in class. The study involved 80 undergraduate students majoring in stomatology. The classroom videos of 5 class hours in the dental anatomy course were collected in October 2022. A quantitative research methodology is used in this study. Pearson correlation, Spearman correlation and multiple linear regression analyses were used to analyze the impact of time and teachers’ linguistic characteristics on students’ concentration.

**Results:**

As a result of regression analysis, the explanatory power of the effect of the linguistic characteristics was 7.09% (F = 83.82, *P* < 0.001), with time, volume and question being significant influencing factors (*P* < 0.01). The local polynomial smooth of the scatter between the concentration degree and the use of questions with time appears to fluctuate cyclically and suggests a potential inverse relationship between the use of questions and the concentration degree.

**Conclusions:**

The results of this study support the significant positive influence of volume and questioning technique, the negative influence of time, and the insignificant influence of speaking speed and the interval between sentences on students’ concentration. This study also suggested that teachers may adjust their questioning frequency based on their observation of students’ concentration.

## Introduction

It is widely acknowledged that students’ classroom concentration on lectures is crucial for achieving better mastery of knowledge. In recent years, an increasing number of factors have served as distractions to students due to the highly digitalized society [1]. Variations in teaching styles among teachers also play an important role in influencing students’ concentration [2, 3].

Although pedagogies such as “problem-based learning (PBL)” and “generated question learning models (GQLM)” have been developed to improve students’ concentration, giving lectures still plays a predominant role in teaching forms [4]. Even under the same subject or based on similar pedagogies, some teachers can be more engaged with students in class, while others may not perform as well. By assessing students’ degree of concentration in response to different teaching characteristics, educators can gain valuable insights into the efficacy of various instructional approaches in promoting students’ concentration.

Evaluating students’ classroom concentration has always been delayed because real-time evaluation can hardly be performed by methods such as self-reporting or testing, while students must “focus” on the lecture instead of judging their own concentrating state. As Bradbury mentioned in 2017, the data supporting the results of how students’ attention changes during a lecture were not satisfactory, as comments made by an individual human observer might not be as accurate as anticipated because of a lack of objectiveness [5]. In Bunce’s research, which was published in 2010, students reported lapses in attention after their occurrence using a clicker [6]. However, what the teacher did when lapses occurred was not considered. It is necessary to assess students’ reactions to the content given by teachers during lectures to determine more specific characteristics of teachers, which may reveal detailed techniques to help teachers give more engaging lectures.

With the help of face recognition technology (FRT), students’ behavior and facial expression can be monitored objectively [7, 8]. In 2023, artificial intelligence face recognition technology was used to evaluate medical students’ classroom concentration in real time [9, 10]. Similar studies focused on both topic and teaching characteristics were performed in 2020, indicating that FRT is a reasonable and reliable tool for detecting student concentration [11]. However, the research presented in this article is insufficient to address our inquiries. Although this study explored the impact of lecture themes through the extraction of keywords via speech recognition, it did not mention the use of pedagogies. Furthermore, this study focused on heuristic classrooms in the field of physics, which demand more critical thinking from students, while memorizing classrooms in the field of medicine requires more understanding and memory from students. Thus, applying similar methods such as FRT and speech recognition technology in medical students’ class concentration remains necessary.

In our study, we transform the lecture recordings into sentences to analyze linguistic characteristics. In 2022, an end-to-end speech recognition toolkit called WeNet was developed. WeNet is a productive toolkit for automatic speech recognition that combines the techniques of an n-gram-based language model, a unified contextual biasing framework and a unified IO system [12]. It is thought to be a productive toolkit with a lower error rate and to support large-scale datasets. WeNet is utilized in this study to accomplish the speech recognition process.

With the help of WeNet, teachers’ linguistic characteristics can be further discussed. Questioning technique is considered as an effective way of promoting students’ concentration in class. A research article about questioning technique in medical education suggested that over half of faculties perceive questioning to be positive for students’ participation in class [13]. However, there is little quantitative evidence proving that the degree to which teachers use questions could significantly enhance students’ instance concentration.

How fast the teacher speaks and how long the teacher pauses during a speech may influence students’ concentration. In 2023, Merhavy studied the influence of lecture playback speed on medical students’ concentration and memory using posttests and indicated that students’ learning ability might not be influenced by faster lecture playback speed [14]. Lenz’s study on teaching strategies suggested that using reflective pauses is beneficial for engaging students in class [13]. Thus, the influence of speaking speed and the interval between sentences are considered in this article.

In Yang’s study, among the extracted audio features, audio volume is a factor that is more related to students’ concentration degree than to their speaking speed [11]. However, little evidence supports a similar conclusion, and further studies are needed to determine the exact relationship between volume and concentration.

Another factor worth considering is the time of the lecture. Bradbury questioned the attention span of students during class, suggesting that more statistical evidence is needed to explain this topic [5]. Thus, this article takes time into consideration and analyses the relationship between the time after the beginning of a lecture and the change in students’ degree of concentration.

In conclusion, with the help of WeNet and FRT, this study will discuss the impact of the pedagogy of using questioning techniques, the speaking speed of the teacher, the volume of the teacher’s voice during the lecture, and the time after the lecture begins on students’ concentration. This study fills the research gap of the specific factors influencing students’ concentration, with a focus on teachers’ speech patterns. It offers valuable insights for educators, inspiring them to adjust their language to enhance students’ engagement in medical classes.

## Materials and methods

### Research design

A quantitative research methodology is used in this study. This study focuses on the relationship between students’ classroom concentration and 5 potentially related factors, including the time after the lecture begins, time interval between sentences, speaking speed, volume, and whether the teacher uses question forms to speak. Based on previous research, this study proposes the following hypotheses: the teacher’s speech pattern will not affect students’ concentration, which includes the speaking speed, the interval time between sentences, the use of questioning techniques, the change of the teacher’s voice volume and the time after the class begins.

### Participants and ethical considerations

In 2022, 80 undergraduate students majoring in stomatology were all included as the research participants, which contains 32 female students and 48 male students. All students were in grade 4, attending the same classes. In order to proceed effectively and efficiently, the convenient sampling method was used in the study. While acknowledging the potential lack of representativeness, this method allows for a deep understanding of the specific cohort, providing valuable insights into the effectiveness of teaching strategies within that particular environment of medical students.

The classroom videos of 5 class hours in the dental anatomy course were collected, and the videos lasted approximately 45 min each. The 5 lectures involved in our study were given by teachers from the same teaching group from department of prosthodontics, including 2 female teachers and 3 male teachers, each gave one lecture to the 80 students individually. In this study, the teachers used consistent lesson plans and teaching methods by delivering lectures through a combination of PowerPoint presentations and oral explanations. The themes of the lectures in this study were dental anatomy, each focusing on different tooth positions. The lesson plans and slides had been produced by the teaching group of the department of prosthodontics, ensuring consistency across different lectures, except for the content being taught.

The study was approved by the Institutional Review Board of Peking University School and Hospital of Stomatology (PKUSSIRB). All students and teachers in the course provided informed consent for this research (Approval number: PKUSSIRB-202274063).

### Data collection

In this study, the FRT, which was proven to be reliable in a previous study, was used to detect students’ facial states and analyze their degree of concentration [9]. A camera (A7S3, Sony, Japan) placed on the lectern is used to record students’ classroom behaviors, which is set to high resolution with a low frame rate (4k, 30fps, 10bit). Each class session lasted for approximately 45 min. The images were exported in chronological order in “.mp4” format.

After converting the “.mp4” files into images using the open-source video conversion software FFmpeg, one frame is extracted per second, resulting in a total of 21,600 images as the dataset for facial detection. The Face ++ facial detection application programming interface (API) is employed to identify facial key points in the images.

### Study parameters

The aim of this study was to focus on teachers’ linguistic characteristics, so sentences were used as the study unit. The characteristics of the sentences and how students’ concentrations changed after the sentences were analyzed. First, the verbal meaning of the sound in the videos was recognized by a speech recognition project called WeNet, an end-to-end speech recognition toolkit [12]. For the following parameters, each sentence was measured separately. Examples of the transcripts are in Table 1.

**Table 1:** Examples of transcripts

The Time after the lecture begins	Speech recognition result (original texture and translation)
02:08.2-02:11.4	牙齿是咀嚼器官的组成之一。
	Teeth are part of the components of the chewing organ.
02:11.8-02:18.8	当然它就是用来吃饭的, 用来嚼碎, 用来咀嚼和切割的实际工具。
	Certainly, they are used as tools for eating, chewing, and cutting the food.
02:19.5-02:25.8	食物进入口腔以后, 刚才说首先要经过切割就是切牙的切割作用。
	After food enters the mouth, it first needs to undergo the cutting process, which is the cutting function of the incisors.
06:54.1-06:59.6	这个位置从牙冠的结构来说, 从外部结构来说, 这个位置是什么?
	In terms of the structure of the crown, what is the name of this position from the perspective of its external structure?

#### Students’ concentration degree

The measurement of students’ concentration is based on FRT [9]. The number of faces judged as “concentrating” by FRT in each image is denoted as SF. The average SF for all the images in each sentence is noted as the “concentration degree (CD)”.

#### The time after the lecture begins

The time after the lecture begins is based on the exact start time of the sentence in the video. The unit is seconds. It is expressed as “time (T)” in this study.

#### Time interval between sentences

This is defined as the time interval between the beginning of a sentence and the end of its former sentence. The unit of the time interval is seconds. It is expressed as “interval (I)” in this study.

#### Speaking speed

Speaking speed represents how fast the teacher speaks. The unit of speaking speed is words (Chinese characters) per second. It is expressed as “speaking speed (SS)”.

#### Volume

In this article, we utilized the AudioSegment class from Python’s pydub package to read audio data and calculate its volume. By leveraging the start and end times of each sentence provided by WeNet, we extracted the audio data accordingly. The AudioSegment class comes with a built-in function for computing the volume of a segment of audio in decibels relative to full scale (dBFS). After reading the audio, the volume of the segment can be directly accessed as a member variable. This parameter is expressed as “volume (V)”.

The volume of a sentence subtracting the volume of the former sentence is noted as the change in volume, which is considered to be another important key characteristic, expressed as “Vc”, with the unit of dBFS as well.

#### Using questioning expression or not

Whether the teacher was using a questioning expression was also recorded. If WeNet decides that the sentence is interrogative, it will be marked as “1”; otherwise, it will be marked as “0”. The parameter is expressed as “question(Q)”.

### Statistical analysis

The collected data were analyzed using the Stata/MP 17.0 program. In this study, Pearson correlation was used for continuous variables, including CD, T, I, SS, V and Vc, and Spearman correlation was used for nominal variables, including Q. Given the complexity of the relationships and the need to control multiple influences simultaneously, linear multiple regression is deemed the most appropriate statistical method for the analysis.

## Results

The number of sentences recognized by WeNet and further taken into account was 3,308. In our analysis, the variable I did not meet the assumptions of normality. The skewness and kurtosis tests revealed significant differences from normality (*P* < 0.05). Following the transformation of the square root of the variable, the distribution of I exhibited improved normality, as assessed by visual inspection and statistical tests. The descriptive statistical results are shown in Table 2.

**Table 2:** Descriptive statistical results

Variable	Obs	Mean	Std. dev.	Min	Max
^**1**^**CD**	3308	8.660	3.022	1.970	22.817
^**2**^**T**	3308	1609.919	940.757	0.000	3494.800
^**3**^**SS**	3308	4.941	1.297	0.378	14.286
^**4**^**V**	3308	-21.624	3.254	-34.645	-15.353
^**5**^**Vc**	3303	0.000	3.115	-14.823	16.920
^**6**^**I**	3303	0.614	0.409	0.000	3.378
^**7**^**Q**	3308	0.182	0.386	0.000	1.000

^1^*CD* concentration degree

^2^*T* time

^3^*SS* speaking speed

^4^*V* volume

^5^*Vc* the change in the volume between a sentence and its previous sentence

^6^*I* interval time between sentences

^7^*Q* whether the teacher used questioning expression or not

The Pearson correlation coefficient was computed to examine the relationships between variables. Correlation analysis revealed a statistically significant negative correlation between CD and T stage (*r* = -0.2036, *P* < 0.001) and a statistically significant positive correlation between CD and V stage (*r* = 0.1717, *P* < 0.001). The correlation analysis also revealed a statistically significant positive correlation between Vc and V (*r* = 0.4779, *P* < 0.001).

Spearman correlation coefficients were calculated to assess the relationships between questions and other variables, with Bonferroni correction for multiple comparisons. The analysis revealed a statistically significant positive correlation between questions and CD (Spearman’s *ρ* = 0.1076, *P* < 0.001) and a marginally significant negative correlation between questions and T (Spearman’s *ρ* = 0.0512, *P* = 0.0677).

As a result of regression analysis, the explanatory power of the effect of the linguistic characteristics was 7.09% (F = 83.82, *P* < 0.001), with T, volume and question being significant influencing factors (*P* < 0.01) (Table 3). We computed the variance inflation factor (VIF) values for each predictor variable in the regression model, and the VIFs were all less than 5 (with a mean VIF of 1.03), indicating that collinearity did not occur within the predictor variables. The linear multiple regression equation is as follows:

**Table 3:** Regression analysis of concentration degree

^1^CD	Coefficient	Std. err.	t	*P* > t	[95% conf. interval]	
^**2**^**T**	− 0.000624	0.0000557	-11.20	0.000	-0.000733	-0.000515
^**3**^**V**	0.141	0.016	8.84	0.000	0.110	0.173
^**4**^**Q**	0.434	0.135	3.22	0.001	0.170	0.698
**_cons**	12.641	0.365	34.66	0.000	11.926	13.356

^1^*CD *concentration degree

^2^*T* time

^3^*V* volume

^4^*Q* whether the teacher used questioning expression or not

CD = -0.000624 T + 0.141 V + 0.434 Q + 12.641

**Fig. 1:**
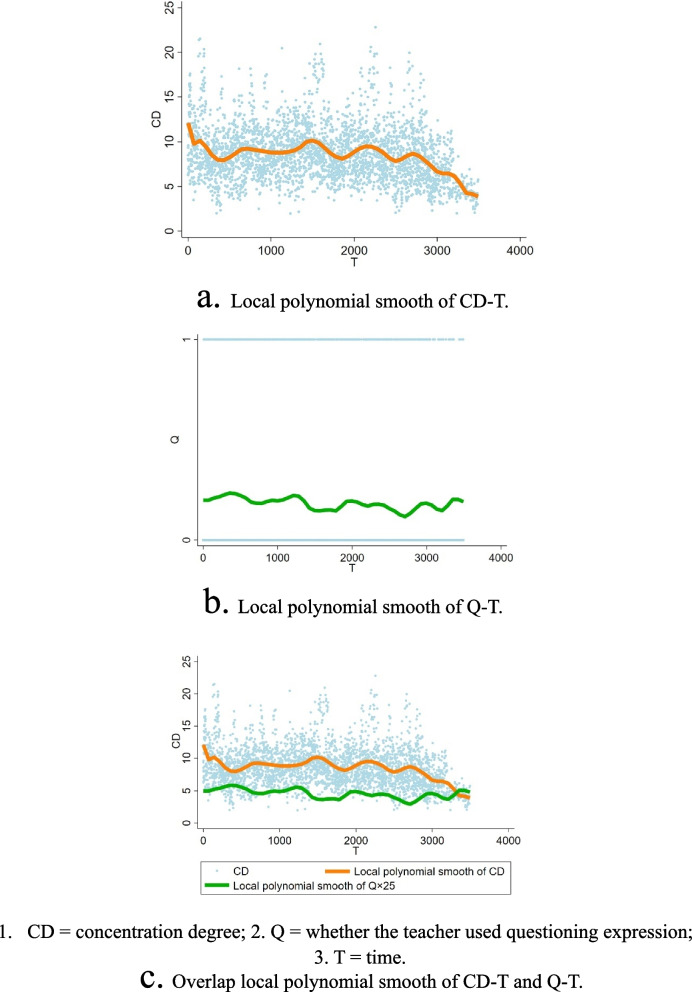
Local polynomial smooth of ^1^CD-^2^T and ^3^Q-T

Due to the observed fluctuating trend in the scatter plot of CD-T in Fig. 1, we generated a local polynomial smooth plot using an Epanechnikov kernel function with a polynomial order of 6. The local smooth polynomials of CD-T and Q-T are depicted in Fig. 1(a) and (b). The overlapping graph is depicted in Fig. 1(c), in which the value of Q is 25 times larger to observe the two curves in one axis jointly.

The local polynomial smooth of the scatter between CD and the question with T appears to fluctuate cyclically akin to that of periodic waves. The cycle of a period is approximately 10–15 min, and the general trend of CD appears to decrease during the lecture.

The local polynomial smooth curves of CD and question appear to exhibit opposite trends, suggesting a potential inverse relationship between them.

## Discussion

In this study on the classroom concentration of medical students, the trends of students’ attention over time and the influence of teachers’ linguistic characteristics on concentration were discussed. The aim was to understand the relationships between various factors and to explore strategies that could improve students’ classroom attention, both individually and collectively.

The design of this study relies on the innovative application of two key technologies. The first one is the speech recognition toolkit called WeNet [12]. It is a great tool for educators to analyze large-scale datasets such as lecture recordings effectively. The other key technology is FRT, which was introduced in 2023 [9]. This study provides further evidence supporting the use of FRT in analyzing students’ concentration.

This study reveals several significant influential factors of teachers’ linguistic characteristics for students’ classroom attention through the analysis of sentences, including time, volume, and questioning techniques. Speaking speed and the interval between sentences and students’ concentration are considered to be nonsignificant influential factors. We also discuss the possible explanations of the curves of the changes in the concentrations of the questions and concentrations with time, which cyclically fluctuate, similar to periodic waves.

The research findings indicate that, akin to common knowledge, students’ classroom concentration tends to decline as the class progresses [13]. Some studies suggest that students’ attention could hardly last for more than 30 min during a lecture [15, 16]. However, in Bunce’s statistical study, students pay attention in a shorter cycle of approximately 4.5 min, and the attention lapse occurs again at a shorter and shorter cycle through the lecture segment [6]. In this study, the relationship between the concentration and time was evidenced by an overall negative correlation between the two variables. According to the results of the scatter plots and polynomial smooth plots, this negative correlation may not be linear. Based on this observation, we conducted a practical exploration and found literature supporting the reproducibility of similar conclusions [5, 17]. Based on the visual results in this study, the cycle of the concentration degree fluctuation period was approximately 10–15 min, with a decreasing trend.

Another correlation finding indicates that volume is also a positive factor influencing concentration. This observation is consistent with previous literature, further corroborating each other’s claims [11].

The statistical insignificance of certain correlations also holds practical significance. Previous research on the impact of video playback speed on students’ attention and memory retention did not find significant relationships, suggesting that playback speeds of ×1.5-2.0 do not interfere with learning outcomes [14, 18]. In this study, the lack of significance between concentration and speaking speed and time interval further supports this conclusion.

In addition, the factors examined in this study included the change in volume from one sentence to its previous sentence. It is commonly assumed that a lecture without variation in volume may be monotonous, while fluctuations in volume might be more engaging. However, this study did not find significant effects of volume change on the concentration. According to the FRT and WeNet results, the impact of the change in volume on the concentration degree is nonsignificant in our study, while the impact of volume is significantly positive.

The results of the local polynomial smooth plots suggest that teachers may engage in unconscious subjective observations of students’ degree of concentration, relying on real-time feedback in their own mind as a potential basis for adjusting their questioning frequency in an attempt to motivate students to increase their level of attentiveness. When students are more focused, teachers may reduce their questioning frequency by employing fewer questioning strategies to advance classroom progress. As presented in the overlapping curves, if there is a causal relationship between the two variables, the response time of this effect should be relatively rapid.

For medical students, attention in memory-based classrooms is more important than in understanding-based classrooms, which is the significance of this study. Most memory-based classes for medical students rely on traditional lecture methods, which are efficient for conveying a large amount of information. However, the passive reception of knowledge may lead to a decline in attention, with students potentially becoming bored and distracted [19]. Some studies suggest that switching courses to an understanding-based format, with more interaction, can increase students’ attention [20]. But under limited conditions, if the course type cannot be changed, then the analysis of teachers’ language characteristics in this study can provide hints for teachers, helping them attract student attention at the linguistic level. The significant results of this study also demonstrate the effectiveness of this possible solution.

This study also has certain limitations. First, according to the results of multiple linear regression, with only 7.09% of the R-squared value indicating that although factors such as time, volume, and the use of questioning have a significant impact on students’ concentration, they do not have a decisive effect. This is understandable because factors such as the type and subject of the course, the use of electronic devices, and the application of teaching methods also have relatively certain influences on classroom concentration1. Due to the basic nature of the speech recognition research method used in this paper, these factors were not fully incorporated into the analysis. Our previous study reported a recall rate of 81.4%, which might have led to the introduction of errors in this study [9]. Therefore, although the FRT was considered reliable in our study, there is still potential for improving its accuracy.

Other parameters of teachers can also affect students’ engagement in class. For example, gender of teacher may have influence on students’ classroom performance. Female teachers may have potential effect on raising female students’ test scores and improve their mental status [21]. The possible explanation of why gender has such influence is that gender could have influence on teachers’ question frequency and expression, which might further lead to the change of students’ classroom engagement [22]. Thus, in this study, questioning techniques were discussed. However, as the FRT used in this study was limited in recognizing the gender of students, relationship between the gender of teachers and students could hardly be fully discussed. More advanced FRT which can count the faces of different genders separately may bring progress on this topic in the future.

In this study, the discussed linguistic parameters represent only a small part of the teacher’s characteristics. As the statistical results show, with an explanatory power of only 7.09%, there are still other factors to be explored in future research. A teacher’s personal charisma has a significant impact on students’ interest and participation in learning [23]. Whether in online or offline courses, traits such as a teacher’s sense of humor, empathy, professional knowledge, and the use of other teaching skills can affect students’ attention in the classroom [24]. However, in this study, the courses used uniform lesson preparation, and the knowledge system was the same, with no special teaching techniques employed by the teachers. Due to the limitations of the artificial intelligent speech recognition toolkit used in this study, the teacher’s sense of humor was not fully assessed. This study has preliminarily confirmed the validity of using toolkits like WeNet in methodology. Discussions on related topics can also be more broadly analyzed in the future with the development of the artificial intelligence technology.

In the future, discussions on other factors affecting classroom attention should continue to expand. This study only conducted a preliminary analysis of the linguistic information of teachers during lectures, with relatively basic factors included. However, in today’s context of promising developments in semantic recognition artificial intelligence, more information related to content meaning rather than simple speech features can also be incorporated into analysis [11]. This may be of significant reference value for teachers in class preparation and classroom strategy design.

## Data Availability

No datasets were generated or analysed during the current study.

## Abbreviations

PBL: Problem-based learning
GQLM: Generated question learning models
FRT: Face recognition technology
API: Application programming interface
dBFS: Decibels relative to full scale
VIF: Variance inflation factor
